# B Cell Synovitis and Clinical Phenotypes in Rheumatoid Arthritis: Relationship to Disease Stages and Drug Exposure

**DOI:** 10.1002/art.41184

**Published:** 2020-03-17

**Authors:** F. Rivellese, F. Humby, S. Bugatti, L. Fossati‐Jimack, H. Rizvi, D. Lucchesi, G. Lliso‐Ribera, A. Nerviani, R. E. Hands, G. Giorli, B. Frias, G. Thorborn, E. Jaworska, C. John, K. Goldmann, M. J. Lewis, A. Manzo, M. Bombardieri, C. Pitzalis, the PEAC‐R4RA Investigators, Iain B. McInnes, Chris Buckley, Peter C. Taylor, Ernest Choy, Arthur Pratt, Christopher Edwards, Nagui Gendi, Pauline Ho, Bhaskar Dasgupta, Patrick Durez, João Eurico Fonseca, Pier Paolo Sainaghi, Mattia Bellan, John Isaacs, Juan D. Cañete, Alberto Cauli, Mattia Congia, Piero Reynolds, Robert Moots, Nora Ng, Carlomaurizio Montecucco, Patrick Verschueren

**Affiliations:** ^1^ Barts and The London School of Medicine and Dentistry Queen Mary University of London London UK; ^2^ IRCCS Policlinico San Matteo Foundation and University of Pavia Pavia Italy; ^3^ Barts Health NHS Trust London UK

## Abstract

**Objective:**

To define the relationship of synovial B cells to clinical phenotypes at different stages of disease evolution and drug exposure in rheumatoid arthritis (RA).

**Methods:**

Synovial biopsy specimens and demographic and clinical data were collected from 2 RA cohorts (n = 329), one of patients with untreated early RA (n = 165) and one of patients with established RA with an inadequate response to tumor necrosis factor inhibitors (TNFi‐IR; n = 164). Synovial tissue was subjected to hematoxylin and eosin and immunohistochemical staining and semiquantitative assessment for the degree of synovitis (on a scale of 0–9) and of CD20+ B cell infiltrate (on a scale of 0–4). B cell scores were validated by digital image analysis and B cell lineage–specific transcript analysis (RNA‐Seq) in the early RA (n = 91) and TNFi‐IR (n = 127) cohorts. Semiquantitative CD20 scores were used to classify patients as B cell rich (≥2) or B cell poor (<2).

**Results:**

Semiquantitative B cell scores correlated with digital image analysis quantitative measurements and B cell lineage–specific transcripts. B cell–rich synovitis was present in 35% of patients in the early RA cohort and 47.7% of patients in the TNFi‐IR cohort (*P* = 0.025). B cell–rich patients showed higher levels of disease activity and seropositivity for rheumatoid factor and anti–citrullinated protein antibody in early RA but not in established RA, while significantly higher histologic synovitis scores in B cell–rich patients were demonstrated in both cohorts.

**Conclusion:**

We describe a robust semiquantitative histologic B cell score that closely replicates the quantification of B cells by digital or molecular analyses. Our findings indicate an ongoing B cell–rich synovitis, which does not seem to be captured by standard clinimetric assessment, in a larger proportion of patients with established RA than early RA.

## INTRODUCTION

The role of B cells in the pathogenesis of rheumatoid arthritis (RA) is well recognized and has been reinforced by the established efficacy of B cell–depleting treatments 1, 2. B cells and B cell effector mechanisms are recognized as a central component of RA synovitis, through local autoantibody production 3, 4, osteoclastogenesis/osteoclast activation 5, 6, and immune complex–mediated inflammatory responses 7, 8. However, the degree of synovial B cell infiltrate is a highly variable phenomenon, ranging from complete absence to a dense distribution within organized infiltrates in up to 40% of patients 9, 10, 11 and as such has been examined as a potential source of predictive and prognostic biomarkers in RA. Indeed, recent data from a large cohort of patients with untreated early RA have suggested that a B cell–rich lymphoid synovitis is associated with highly active disease and predictive of radiographic progression 12. However, comparison of these data with other cohorts that either support 13, 14 or refute 15, 16, 17 this notion is challenging due to a lack of consistency in quantitative and qualitative assessment of B cell synovitis, the effects of concomitant diverse therapy, and examination of patients at variable disease stages and levels of disease activity.

Importantly, it remains unclear whether an association between B cell synovitis and disease severity is modulated during disease progression, and moreover, whether the prevalence of B cell synovitis remains stable or is enriched through disease evolution and/or cycles of nonresponse to therapy. Since 2008, the Centre for Experimental Medicine and Rheumatology at Queen Mary University of London (QMUL) has been collecting pretreatment synovial tissue as part of a pathobiology‐driven patient stratification program in RA. This program includes patients enrolled in 2 multicenter precision‐medicine clinical studies collecting pretreatment synovial tissue at specific disease stages: untreated early RA (the Pathobiology of Early Arthritis Cohort [PEAC; http://www.peac-mrc.mds.qmul.ac.uk]) and established RA in patients with an inadequate response to tumor necrosis factor inhibitors (TNFi‐IR) (Response–Resistance to Rituximab versus Tocilizumab in RA [R4RA; http://www.r4ra-nihr.whri.qmul.ac.uk]).

In this study, we developed and validated a semiquantitative scoring system focused on the measurement of B cell synovitis in RA. By analyzing the prevalence of B cell synovitis using a robust histologic score, we examined whether the association between B cell synovitis and clinical phenotype is a stable phenomenon during disease evolution or is enriched in a cohort of patients with treatment‐resistant established RA.

## PATIENTS AND METHODS

#### Patients

A total of 329 patients fulfilling the 2010 American College of Rheumatology/European League Against Rheumatism classification criteria for RA 18 were evaluated at 2 disease stages: early RA and established RA with an inadequate response to TNFi. The early RA cohort consisted of 165 consecutive patients with untreated early RA (disease duration <1 year) recruited as part of the Medical Research Council–funded multicenter PEAC. The established RA cohort consisted of 164 patients with an inadequate response to TNFi from the multicenter R4RA trial. The PEAC‐R4RA investigators are listed in Appendix A.

Synovial tissue specimens were obtained from all patients at study entry by ultrasound (US)–guided synovial biopsy 19 or arthroscopic approaches depending on the expertise of the local recruiting center. A minimum of 6 samples for subsequent histologic analysis and 6 samples for RNA extraction were retained from each procedure. Patient demographic characteristics and clinical parameters collected at baseline included sex, disease duration, anti–citrullinated protein antibody (ACPA) and rheumatoid factor (RF) status, erythrocyte sedimentation rate (ESR), C‐reactive protein (CRP) level, swollen and tender joint counts, global health score (measured on a visual analog scale), and concomitant medications. All patients provided written informed consent, and each study received local ethics approval (PEAC LREC: 05/Q0703/198; R4RA: LREC 12/WA/0307).

#### Semiquantitative histopathologic scoring and digital image analysis

Formalin‐fixed and paraffin‐embedded synovial tissue sections (3 μm) were stained with hematoxylin and eosin (H&E) and semiquantitatively assessed for the degree of synovitis (on a scale of 0–9), according to a previously validated score 20. Consecutive sections were subjected to immunohistochemical staining for CD20 (mouse IgG2a anti‐human CD20) (clone L26; Dako), CD68 (mouse IgG1 anti‐human CD68) (clone KP1; Dako), and CD138 (mouse IgG1 anti‐human CD138) (clone MI15; Dako), as previously described 13. Slides were counterstained with hematoxylin and mounted. Images were acquired on an Olympus microscope using cellSens software.

**Figure 1:**
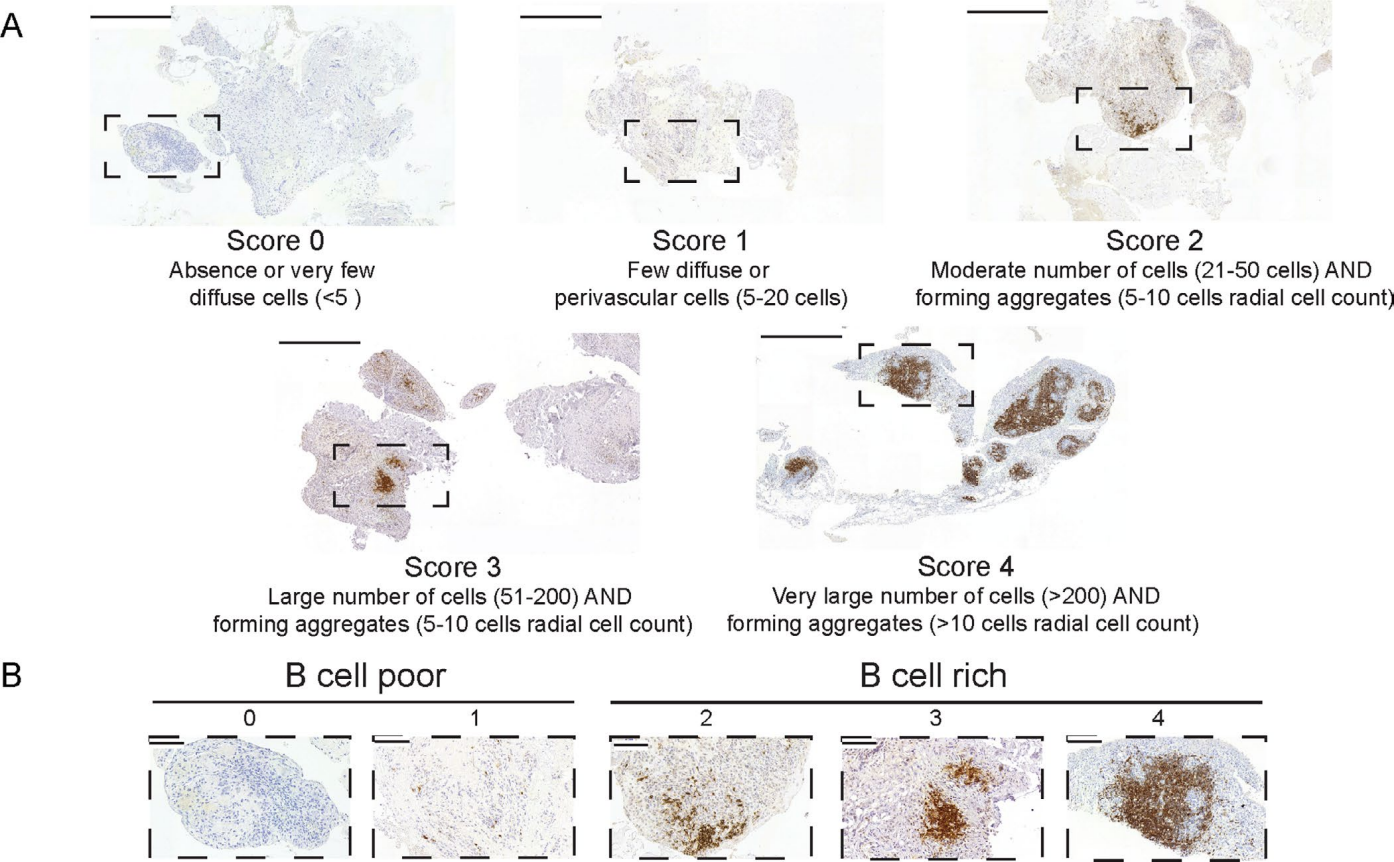
B cell scoring. **A**, Representative images for each semiquantitative CD20 immunohistochemical score in patients with rheumatoid arthritis with an inadequate response to tumor necrosis factor inhibitors. Bars = 500 μm. **B**, Higher‐magnification views of the boxed areas in **A**. Bars = 100 μm.

First, the nature and quality of the tissue biopsy specimens were assessed by evaluating tissue morphology by H&E staining and macrophage distribution (CD68 staining). To be considered suitable for further histologic analysis, synovial samples had to present either a clear synovial lining (CD68+ cells in a linear arrangement) or sublining (characteristic vessels and stroma). Samples were rejected and classified as ungraded if there was no recognizable synovial tissue or there were artifactual changes (i.e., tissue folds, cutting and staining artifacts) in immunohistochemical stainings. Samples with synovium were scored semiquantitatively (on a scale of 0–4) for the degree of CD20+ B cell infiltration, adapting a previously described B cell aggregation score 21 (Figures 1A and B). Patients were classified as B cell poor (semiquantitative score 0–1) or B cell rich (score 2–4), as shown in the flow chart in Supplementary Figure 1, available on the *Arthritis & Rheumatology* web site at http://onlinelibrary.wiley.com/doi/10.1002/art.41184/abstract. In B cell–poor samples, the presence of plasma cells was assessed, and samples with a semiquantitative CD138 score of ≥2 underwent an additional staining for CD20 at a deeper cutting level for a final classification as B cell poor or B cell rich. An overview of the cutting protocol at different levels is shown in Supplementary Figure 2, available on the *Arthritis & Rheumatology* web site at http://onlinelibrary.wiley.com/doi/10.1002/art.41184/abstract. All samples were assessed by 2 independent observers.

For quantitative analysis using digital image analysis, whole CD20‐stained slides were reacquired using a Panoramic 250 High‐Throughput Scanner (3DHISTECH). Images were visualized and analyzed on Fiji software 22 using a pipeline developed in‐house (by DL) to obtain the following measurements: total stained area, total tissue area, and area fraction (percent of stained area, calculated as total stained area/total tissue area × 100). In the digital image analysis pipeline, the operator first manually selected region(s) of interest corresponding to the synovial tissue detectable on the slide (the “total tissue area”). The script then 1) removed the areas outside the region(s) of interest, 2) performed a color deconvolution to isolate the diaminobenzidine (DAB) color vector (method “H‐DAB”), and 3) calculated the intensity threshold (method “Default,” manually adjusted if necessary) on the DAB channel. Finally, the software calculated the area included in the threshold (“total stained area”). An example of digital image analysis is shown in Supplementary Figure 3, available on the *Arthritis & Rheumatology* web site at http://onlinelibrary.wiley.com/doi/10.1002/art.41184/abstract.

In a pilot assessment exercise, digital image analysis was performed in parallel in a blinded manner by 2 observers from 2 different centers (QMUL and University of Pavia). The analysis was performed on 15 random samples from the early RA PEAC cohort and replicated on the first 100 consecutive samples from the TNFi‐IR R4RA cohort in order to obtain independent measurements. Intraclass correlation coefficients for the level of agreement between the 2 observers and Bland‐Altman plots were generated. Following this validation exercise, digital image analysis was performed in the subset of patients from the early RA cohort who underwent RNA sequencing (n = 91) and in the patients in the TNFi‐IR cohort who had graded synovial tissue samples (n = 155).

#### RNA extraction and RNA sequencing

RNA extraction and RNA sequencing were performed as previously described 12 on synovial tissue samples from 94 patients in the early RA PEAC cohort and 128 patients in the TNFi‐IR R4RA cohort for whom RNA was available. For the PEAC cohort, RNA was extracted from synovial tissue homogenized at 4°C in TRIzol reagent, according to the recommendations of the manufacturer (ThermoFisher Scientific, Invitrogen Division), followed by a phenol–chloroform extraction. Briefly, the tissue lysate was mixed vigorously with chloroform before centrifugation. The aqueous phase was removed and mixed with ice‐cold isopropanol for 30 minutes. After further centrifugation, the RNA pellet was washed in 70% ethanol before air‐drying and resuspension in RNase‐free water.

The concentration/purity of RNA samples was measured using a NanoDrop 2000c spectrophotometer (LabTech) and Qubit assay (ThermoFisher Scientific, Invitrogen Division). RNA quality (RNA integrity number) was assessed using an Agilent 2100 Bioanalyzer or Agilent TapeStation system. One microgram of total RNA was used as input material for library preparation using a TruSeq RNA Sample Preparation Kit v2 (Illumina). Generated libraries were amplified with 10 cycles of polymerase chain reaction (PCR). The size of the libraries was confirmed using a 2200 TapeStation system and High‐Sensitivity D1K ScreenTape (Agilent Technologies), and their concentration was determined by a quantitative PCR (qPCR)–based method using a Library Quantification kit (KAPA). The libraries were first multiplexed (5 per lane) and then sequenced on an Illumina HiSeq 2500 system to generate 50 million paired ends with 75 base pair reads.

For the R4RA cohort, the RNA extraction method was the same as for the PEAC, but subsequent to homogenization in TRIzol, a column‐based extraction (Zymo Direct‐zol RNA MicroPrep) was used according to the recommendations of the manufacturer for the majority of samples (n = 124), while phenol–chloroform extraction as described above was used on a small number of samples (n = 4). Principal components analysis confirmed that there were no major differences between the 2 extraction methods (data not shown). One hundred fifty to five hundred nanograms of total RNA was used as input material for library preparation using NEBNext Ultra RNA Library Prep Kit for Illumina according to the recommendations of the manufacturer (New England Biolabs). Generated libraries were amplified with 13 cycles of PCR. Library quality control was performed with MiSeq Nano QC, and the final concentration was determined by a qPCR‐based method using a Library Quantification kit. The libraries were first multiplexed and then sequenced on an Illumina HiSeq 4000 system to generate 50 million paired ends with 150 base pair reads (Genewiz). A summary of the RNA extraction and sequencing methods for the 2 cohorts is included in Supplementary Table 1, available on the *Arthritis & Rheumatology* web site at http://onlinelibrary.wiley.com/doi/10.1002/art.41184/abstract.

#### RNA sequencing analysis

For the PEAC cohort, transcript abundance was derived from fastq files over Gencode version24/GRCh38 transcripts using Kallisto version 0.43.0 23. Transcript abundances and average transcript lengths were imported into R using tximport 1.10.0. Imported abundances were normalized in R, including a correction for average transcript length and incorporating batch, sex, and synovial histology patterns (pathotypes) 12 as model covariates, using DESeq2 1.22.0 24. Transcript abundances underwent regularized log expression (RLE) transformation. The RNA‐Seq data have been deposited in the ArrayExpress database (online at https://www.ebi.ac.uk/arrayexpress; accession no. E‐MTAB‐6141). Outliers were identified and removed using principal components analysis, leaving 91 samples for further analysis.

For the R4RA cohort, transcript abundance was quantified using Salmon version 0.12.0 25 with the Gencode version 29/GRCh38 annotation, and quantifications were aggregated to genes using tximport version 1.10.1. Data were subjected to variance‐stabilizing transformation to remove the dependency of the variance on the count mean using DESeq2 version 2.16.1 24. Outliers were identified and removed using principal components analysis, leaving 127 samples for further analysis.

#### Definition of the B cell–specific gene module

Cell‐specific gene modules were obtained as previously described 26. Briefly, we downloaded RLE‐normalized FANTOM5 data from http://fantom.gsc.riken.jp/5/data/. Upon selecting primary cells and tissue and excluding derived cells, stimulated cells and cell line data were Z score–normalized, and the expression of each gene was ranked across all tissues and cells. A specificity score was determined by counting the number of tissues and cells showing increased gene expression (Z score >3 [>3 SD above the mean across all tissues]), so that the most tissue‐specific genes would have the lowest specificity scores. Genes were considered specific to a tissue type or cell type when: 1) the level of gene expression was in the top 3 tissues (i.e., rank 1–3); 2) the Z score was >5 (i.e., >5 SD above the mean expression across all tissues); and 3) the specificity score was <10 tissues. Gene modules for different cell types were consistent with lists of genes previously published by the FANTOM5 consortium for several cell types 27, 28. The list of B cell–specific genes (B cell module; including Z scores, specificity scores, and rank) is shown in Supplementary Table 2, available on the *Arthritis & Rheumatology* web site at http://onlinelibrary.wiley.com/doi/10.1002/art.41184/abstract.

#### Synovial RNA‐Seq B cell module scores

The B cell module was used to calculate synovial RNA‐Seq B cell module scores, by applying singular value decomposition to synovial RNA‐Seq data 29. Briefly, the normalized gene expression matrix for the patients was filtered to include only the B cell module genes. Next, the matrix was subjected to singular value decomposition, and the first principal component score was taken as the module expression or “module score” for plotting. B cell module scores were analyzed for correlation against histologic markers in synovial tissue using Spearman's correlation.

#### Statistical analysis

Measures of central tendency and dispersion and statistical tests used are indicated in each figure legend. Generally, the following statistical tests were used: Mann‐Whitney test for comparison between 2 groups, one‐way analysis of variance with Bonferroni post hoc adjustment for comparison between multiple groups, chi‐square test for proportions, and Spearman's test for correlations. Statistical analyses were performed using IBM SPSS version 20 and RStudio version 0.99.486. *P* values less than 0.05 were considered significant.

## RESULTS

#### Demographic and clinical features of the patients with early RA and those with established RA

A total of 329 patients with RA were included in this study: 165 with untreated early RA (PEAC) and 164 with an inadequate response to TNFi (R4RA trial). Table 1 summarizes the patient characteristics. Both cohorts included patients with highly active RA (mean Disease Activity Score in 28 joints [DAS28] >5.1), with no differences in terms of disease activity (DAS28 and its components) and other clinical features, except for a higher prevalence of female patients, higher CRP levels, and higher proportion of ACPA‐positive patients in the cohort with established RA. There were no differences between the cohorts with regard to the type of joint biopsied. Importantly, >70% of the samples were obtained from small joints (i.e., wrists or metacarpophalangeal joints), and >90% of the procedures were performed under US guidance. The procedures had an excellent success rate in terms of retrieval of gradable tissue, ranging from 86.7% (143 of 165) in the early RA cohort to 94.5% (155 of 164) in the TNFi‐IR cohort. These results demonstrate that both the early RA and established RA cohorts included patients with active RA, although the patients with established RA showed more aggressive features, such as a greater proportion of patients with autoantibody positivity and higher levels of markers of inflammation, which is compatible with their recruitment after anti‐TNF failure.

**Table 1:** Characteristics of the patients in the early RA and TNFi‐IR cohorts^a^

	Early RA (n = 165)	TNFi‐IR (n = 164)	*P* b
Age, years	53.2 ± 15.3	54.6 ± 13.3	NS
Sex, % female	66.1	79.9	0.005
Disease duration, years	0.5 ± 0.6	12.8 ± 10.8	<0.001
DAS28	5.7 ± 1.4	5.63 ± 1.27	NS
Tender joint count	11.6 ± 7.6	11.85 ± 7.8	NS
Swollen joint count	7.5 ± 5.6	6.92 ± 5.10	NS
Global health score (100‐mm VAS)	61.5 ± 26.9	66.33 ± 24.99	NS
ESR, mm/hour	37.8 ± 28.4	34.47 ± 25.95	NS
CRP, mg/liter	17.6 ± 27.1	21.13 ± 27.97	0.01
ACPA positive, %^c^	64.2	75.3	0.03
RF positive, %	65.5	71.4	NS
No. of conventional synthetic DMARDs received, %			NA
0	100	2.4	
1	0	67.7	
2	0	21.3	
3	0	8.4	
Receiving steroids at the time of biopsy, %	0	42.2	NA
Joint biopsied, %			NS
Wrist	64.8	60.4	
Knee	18.2	23.8	
MCP	13.9	11	
Other	3.1	4.8	
Joint size, %			NS
Large	25.2	27.4	
Small	74.8	72.6	
Synovial sampling technique, %			NA
US‐guided	100	86	
Arthroscopy	0	14	

a Except where indicated otherwise, values are the mean ± SD. RA = rheumatoid arthritis; TNFi‐IR = inadequate response to tumor necrosis factor inhibitors; NS = not significant; DAS28 = Disease Activity Score in 28 joints; VAS = visual analog scale; ESR = erythrocyte sedimentation rate; CRP = C‐reactive protein; RF = rheumatoid factor; DMARDs = disease‐modifying antirheumatic drugs; NA = not applicable; MCP = metacarpophalangeal; US = ultrasound.

b By chi‐square test or Mann‐Whitney test.

c Anti–citrullinated protein antibodies (ACPAs) were measured using a clinically available standard pathology laboratory anti–cyclic citrullinated peptide antibody 2 assay.

#### Validation of semiquantitative synovial B cell scoring by digital image analysis

**Figure 2:**
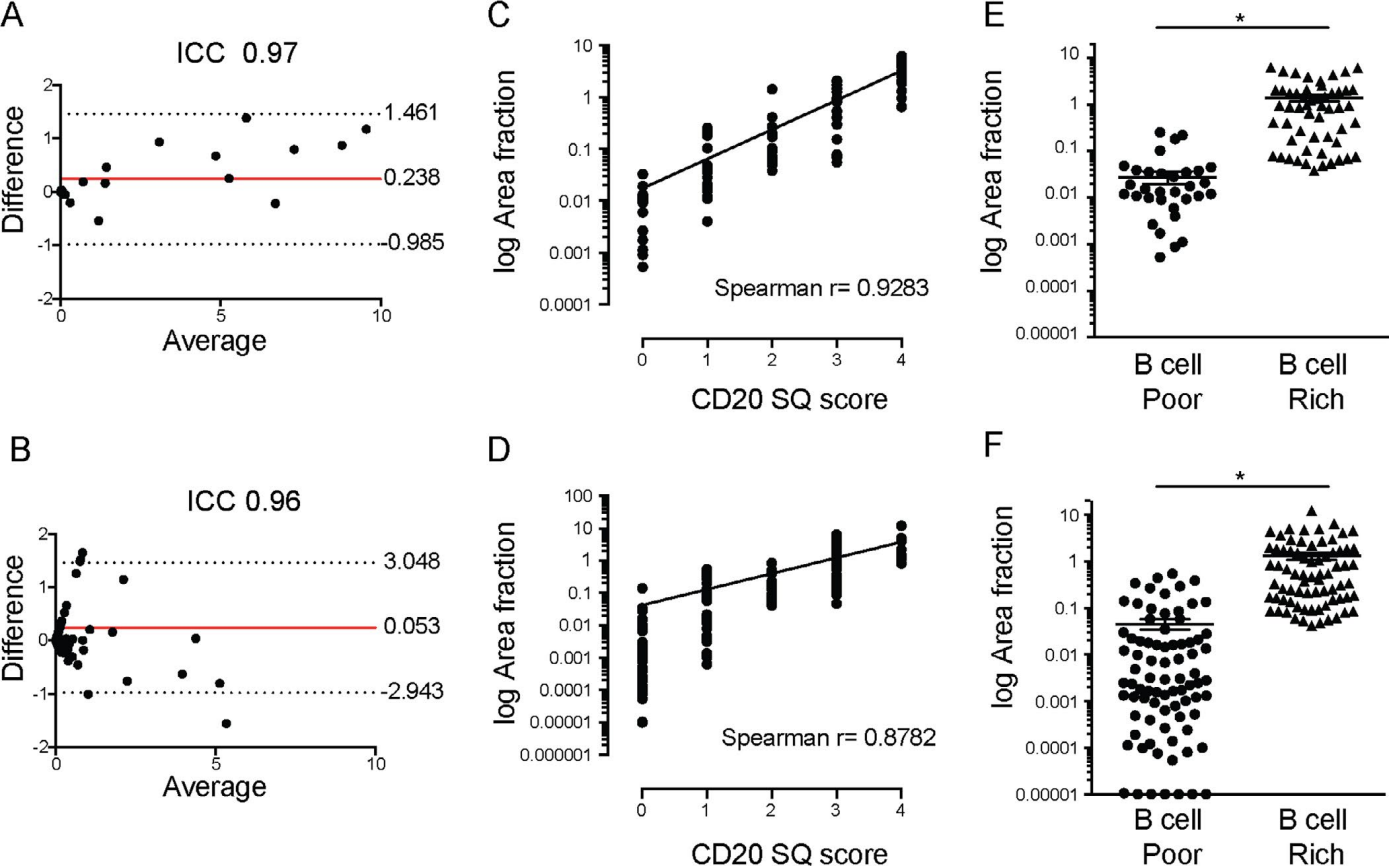
Semiquantitative (SQ) B cell scores and digital image analysis. **A** and **B**, Bland‐Altman plots showing the difference between and average of 2 measurements of CD20+ B cell area fraction (percent of stained area, calculated as total stained area/total tissue area × 100) in the cohort of patients with early rheumatoid arthritis (RA; n = 15) (**A**) and in the cohort of patients with established RA with an inadequate response to tumor necrosis factor inhibitors (TNFi‐IR; n = 100) (**B**), obtained by 2 independent observers in 2 different centers (Queen Mary University of London and University of Pavia). Solid lines indicate the mean; dotted lines indicate the 95% confidence interval. ICC = intraclass correlation coefficient. **C** and **D**, Correlation between semiquantitative CD20+ B cell score and CD20+ B cell area fraction in the early RA cohort (n = 91) (**C**) and the TNFi‐IR cohort (n = 155) (**D**). **E** and **F**, CD20+ B cell area fraction in the early RA cohort (n = 91) (**E**) and the TNFi‐IR cohort (n = 155) (**F**) classified as B cell poor or B cell rich. Symbols represent individual patients; horizontal lines and error bars show the mean ± SD. * = *P* < 0.05 by Mann‐Whitney test. Color figure can be viewed in the online issue, which is available at http://onlinelibrary.wiley.com/doi/10.1002/art.41184/abstract.

To quantify the presence of B cells in synovia, we adapted a semiquantitative scoring method (scale of 0–4) previously described by our group 21. Representative examples of tissue samples for each score are shown in Figures 1A and B, and a flow chart of the evaluation of the biopsy samples is shown in Supplementary Figure 1, available on the *Arthritis & Rheumatology* web site at http://onlinelibrary.wiley.com/doi/10.1002/art.41184/abstract. In order to validate the semiquantitative B cell scores against an objective measurement, we used computer‐aided automated digital image analysis. To this end, whole slide images were acquired, and digital image analysis was used to obtain the following measurements: total tissue area (in μm^2^), total stained area, and area fraction (percent of stained area, calculated as total stained area/total tissue area × 100). An example of the digital image analysis approach is shown in Supplementary Figure 3, available on the *Arthritis & Rheumatology* web site at http://onlinelibrary.wiley.com/doi/10.1002/art.41184/abstract. First, we aimed to evaluate the reproducibility of digital image analysis, by performing the analyses in 2 different centers (QMUL and University of Pavia) in a blinded manner on 15 randomly selected synovial samples from the early RA cohort and 100 consecutive samples from the TNFi‐IR cohort. These analyses showed an excellent agreement rate, with an intraclass correlation coefficient of 0.97 in the early RA cohort (Figure 2A) and 0.96 in the TNFi‐IR cohort (Figure 2B).

Having confirmed the reproducibility of digital image analysis, we next aimed to evaluate the correlation between the semiquantitative and digital image analysis (CD20+ area fraction) histologic B cell scores in 91 patients in the early RA cohort and 164 patients in the TNFi‐IR cohort. Our results demonstrated a strong correlation between the semiquantitative and digital image analysis CD20 scores (Spearman's r = 0.93 in the early RA cohort and 0.88 in the TNFi‐IR cohort; *P* < 0.0001) (Figures 2C and D). In addition, we determined the mean CD20+ area fraction in patients classified as B cell rich and those classified as B cell poor according to the semiquantitative scores and demonstrated a significantly higher CD20+ area fraction in B cell–rich patients, as shown in Figures 2E and F (mean ± SD area fraction 1.4 ± 1.6 in B cell–rich patients and 0.02 ± 0.05 in B cell–poor patients in the early RA cohort [*P* < 0.0001] and 1.3 ± 1.9 in B cell–rich patients and 0.04 ± 0.1 in B cell–poor patients in the TNFi‐IR cohort [*P* < 0.0001]). Overall, our results demonstrate that the semiquantitative CD20 score presented both as a raw score and when applied to stratify patients into B cell–rich and B cell–poor cohorts reliably reflects quantitative measures of CD20 B cell infiltration assessed using computer‐aided digital image analysis.

#### Correlation between semiquantitative and quantitative histologic scores and B cell–lineage genes

**Figure 3:**
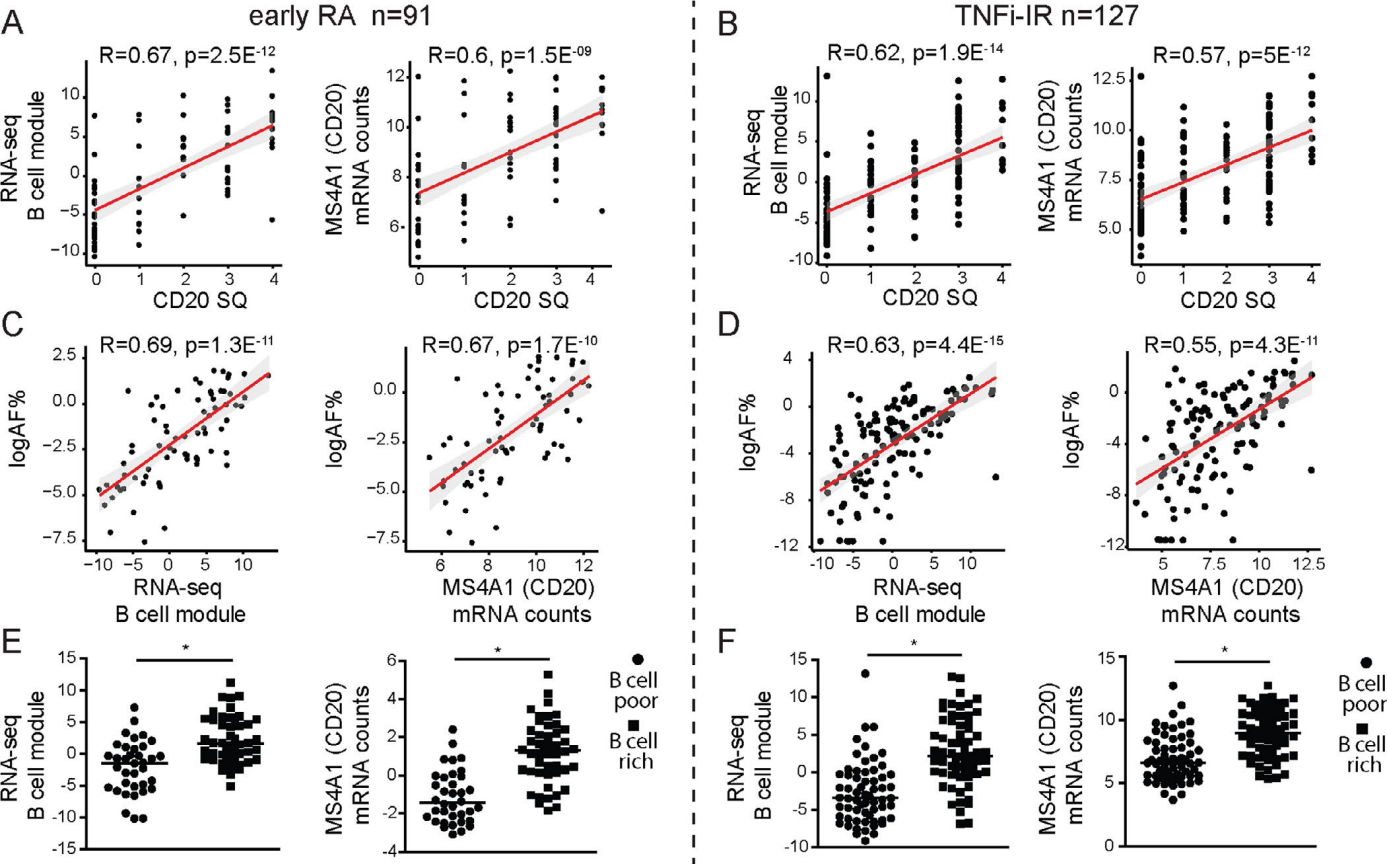
Correlation of semiquantitative (SQ) B cell scores with RNA‐Seq B cell module scores. **A** and **B**, Correlations between the semiquantitative CD20+ B cell score and the RNA‐Seq B cell module and between the semiquantitative CD20+ B cell score and CD20/MS4A1 gene expression levels in the cohort of patients with early rheumatoid arthritis (RA) (**A**) and the cohort of patients with established RA with an inadequate response to tumor necrosis factor inhibitors (TNFi‐IR) (**B**). **C** and **D**, Correlations between the CD20+ B cell area fraction (AF) (percent of stained area, calculated as total stained area/total tissue area × 100) and the RNA‐Seq B cell module and between the CD20+ B cell area fraction and CD20/MS4A1 gene expression levels in the early RA cohort (**C**) and the TNFi‐IR cohort (**D**). In **A**–**D**, symbols represent individual patients (n = 91 for early RA and 127 for TNFi‐IR); Lines and shading indicate the regression line and 95% confidence interval. **E** and **F**, RNA‐Seq B cell module and CD20/MS4A1 gene expression levels in B cell–poor and B cell–rich patients in the early RA cohort (n = 91) (**E**) and TNFi‐IR cohort (n = 127) (**F**). Symbols represent individual patients; horizontal lines show the mean. * = *P* < 0.05 by Mann‐Whitney test. Color figure can be viewed in the online issue, which is available at http://onlinelibrary.wiley.com/doi/10.1002/art.41184/abstract.

Next, we evaluated the relationship between CD20+ B cell histologic scores (semiquantitative and digital image analysis) and B cell–specific gene expression levels. To this end, we correlated histologic scores for B cells with CD20 gene expression levels and RNA‐Seq B cell module scores obtained by RNA sequencing of synovial tissue from 91 patients in the early RA cohort and 127 patients in the TNFi‐IR cohort. Our results demonstrated a strong correlation between the semiquantitative CD20 score and B cell module score and between the semiquantitative CD20 score and CD20 gene expression, both in the early RA cohort (Figure 3A) and the TNFi‐IR cohort (Figure 3B). Similarly, we observed a positive correlation between the digital image analysis CD20 area fraction and the B cell module score and between the digital image analysis CD20 area fraction and CD20 gene expression in the early RA cohort (Figure 3C) and the TNFi‐IR cohort (Figure 3D). Finally, when patients were segregated into B cell–rich and B cell–poor subgroups, we demonstrated significantly higher levels of B cell module scores and CD20 gene expression in B cell–rich patients in both cohorts (Figures 3E and F). Overall, our results demonstrate that the semiquantitative CD20 score presented both as a raw score and when applied to stratify patients into B cell–rich and B cell–poor cohorts accurately reflects the levels of synovial B cell gene expression.

#### Clinical features of B cell–rich patients and B cell–poor patients at different disease stages

**Figure 4:**
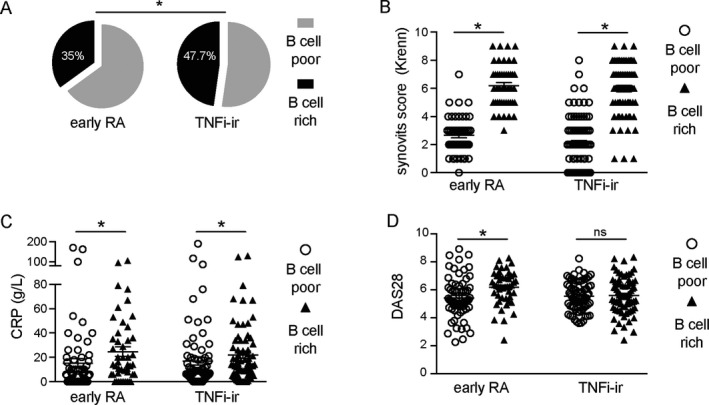
B cell–rich synovitis at different rheumatoid arthritis (RA) disease stages. **A**, Prevalence of B cell–rich synovitis in the cohort of patients with early RA and in the cohort of patients with established RA with an inadequate response to tumor necrosis factor inhibitors (TNFi‐IR). **B**–**D**, Krenn synovitis score (**B**), C‐reactive protein (CRP) level (**C**), and Disease Activity Score in 28 joints (DAS28) using the erythrocyte sedimentation rate (**D**) in B cell–poor and B cell–rich patients in the early RA cohort and the TNFi‐IR cohort. Symbols represent individual patients (n = 143 for early RA and 155 for TNFi‐IR); horizontal lines and error bars show the median and interquartile range in **B** and the mean ± SD in **C** and **D**. * = *P* < 0.05 by chi‐square test in **A**; by Mann‐Whitney test in **B**–**D**. NS = not significant.

Next, we applied the semiquantitative CD20 score and algorithm shown in Figure 1 and Supplementary Figure 1 to stratify patients from the 2 cohorts into B cell–rich and B cell–poor categories. We assessed the prevalence of B cell–rich synovitis at different stages of disease evolution and drug exposure, i.e., in untreated early RA versus established RA with an inadequate response to TNFi. Interestingly, we demonstrated a significantly higher prevalence of B cell–rich synovitis in patients with established RA with an inadequate response to TNFi (47.7% in the TNFi‐IR cohort and 35% in the untreated early RA cohort; *P* = 0.025) (Figure 4A), suggesting an increase in the proportion of B cell–rich samples in longstanding treatment‐resistant RA.

Furthermore, we examined the relationship between clinical phenotype and B cell–rich synovitis at each disease stage (Table 2). Consistent with previously published data 12, compared to B cell–poor patients, patients with B cell–rich synovitis in the early RA cohort showed higher levels of synovial inflammation, as measured by the Krenn synovitis score (*P* < 0.0001), which is a well‐validated composite score measuring the enlargement of the lining cell layer, the cellular density of synovial stroma, and leukocyte infiltrate. Accordingly, B cell–rich patients had significantly higher levels of other immune cells (T cells, macrophages, and plasma cells). Clinically, B cell–rich patients showed higher disease activity (*P* = 0.005 for DAS28 and *P* = 0.048 for swollen joint count), markers of inflammation (*P* = 0.001 for ESR and *P* = 0.012 for CRP level), and a higher prevalence of autoantibody positivity (*P* = 0.024 for ACPA and *P* = 0.023 for RF) than B cell–poor patients.

**Table 2:** Clinical phenotype of B cell–poor versus B cell–rich patients at different RA disease stages^a^

	Early RA(n = 143)			TNFi‐IR(n = 155)		
	B cell poor (n = 93 [65.0%])	B cell rich (n = 50 [35.0%])	*P* b	B cell poor (n = 81 [52.3%])	B cell rich (n = 74 [47.7%])	*P* b
DAS28	5.6 ± 1.38	6.1 ± 1.2	0.005	5.6 ± 1.3	5.7 ± 1.2	NS
Tender joint count	11.3 ± 7.3	13 ± 7.7	NS	12.5 ± 7.9	11.2 ± 7.8	NS
Swollen joint count	7.1 ± 5.4	8.8 ± 5.7	0.048	6.5 ± 5.1	7.4 ± 5.1	NS
Global health score (100‐mm VAS)	60.6 ± 28.6	64.8 ± 25.2	NS	65.2 ± 23.2	66.8 ± 27.3	NS
ESR, mm/hour	34.6 ± 28.9	49.5 ± 28.9	0.001	31.7 ± 24.4	38.3 ± 27.2	NS
CRP, mg/liter	17.5 ± 32.7	21.5 ± 24.2	0.012	19.9 ± 35.9	26.4 ± 26.8	0.001
ACPA positive, %^c^	59.8	78.4	0.024	73.1	76.4	NS
RF positive, %	62	80.4	0.023	70	72.2	NS
No. of conventional synthetic DMARDs received, %			NA			NS
0	100	100		1.2	4.1	
1	0	0		70.4	67.6	
2	0	0		21	23	
3	0	0		7.4	5.4	
Receiving steroids at the time of biopsy, %	0	0	NA	39.7	41.9	NS
Synovitis score	3 ± 1	6 ± 1	<0.0001	2 ± 2	6 ± 2	<0.0001
Semiquantitative CD3 score ≥2, %	6.5	70.8	<0.0001	9.1	90.9	<0.0001
Semiquantitative CD68L score ≥2, %	12.2	46	<0.0001	6.2	93.8	<0.0001
Semiquantitative CD68SL score ≥2, %	24.2	84	<0.0001	17.1	82.9	<0.0001
Semiquantitative CD138 score ≥2, %	4.4	78	<0.0001	9.3	90.7	<0.0001

a Patients with ungraded synovial biopsy samples were excluded. Except where indicated otherwise, values are the mean ± SD. CD68L = CD68 lining; CD68SL = CD68 sublining (see Table 1 for other definitions).

b By Mann‐Whitney test or Fisher's exact test.

c ACPAs were measured using a clinically available standard pathology laboratory anti–cyclic citrullinated peptide antibody 2 assay.

Conversely, in the cohort of patients with established disease (TNFi‐IR), while the significant association of B cell–rich synovitis with histologic synovitis and immune cell infiltration was maintained (Table 2 and Figure 4B), the only significant difference in terms of systemic inflammation or disease activity was a higher CRP level in B cell–rich patients than in B cell–poor patients (Table 2 and Figure 4C) (*P* = 0.001). There were no differences in terms of disease activity as measured by DAS28 (Figure 4D) and its components (Table 2). Importantly, there were also no differences in the use of synthetic disease‐modifying antirheumatic drugs (DMARDs) or steroids between B cell–rich and B cell–poor patients. These data suggest that the clinical phenotype of highly active, aggressive RA associated with a B cell–rich synovitis at disease initiation is subsequently lost with disease progression and/or modulation of synovial pathobiology by concomitant therapy.

To further explore the association of synovial histopathology with clinical phenotypes, we compared clinical features in patients classified according to semiquantitative scores for T cells, macrophages, and plasma cells ([Link], [Link], [Link], [Link], available on the *Arthritis & Rheumatology* web site at http://onlinelibrary.wiley.com/doi/10.1002/art.41184/abstract). In the early RA cohort, higher levels of markers of inflammation were observed in patients with higher infiltration of all immune cells. On the contrary, a higher prevalence of autoantibody positivity was observed only in patients who, in addition to B cells, were rich in synovial T cells and plasma cells, but no relationship was observed with CD68L or CD68SL macrophage populations. Notably, on the other hand, higher disease activity measured by DAS28 was present only in patients with higher sublining macrophages and plasma cell infiltration. In the cohort of patients with established RA, patients with synovitis rich in T cells, sublining macrophages, and plasma cells showed significantly higher CRP levels, while there were no differences in any other clinical parameter. These data add new insights into the association of immune cells with specific disease features, while confirming a lack of association between synovitis and clinical phenotypes in the cohort of patients with established RA.

## DISCUSSION

In this study, we explored the prevalence and relationship to clinical phenotypes of B cell synovitis in a large, well‐characterized cohort of 329 patients at 2 different stages of disease evolution and drug exposure. We validated and applied a semiquantitative B cell synovitis score that accurately reflects quantitative measures of both cellular and molecular B cell infiltrate, and demonstrated that the prevalence of B cell synovitis was enriched in patients with treatment‐resistant established disease. Additionally, although patients with established RA display a more severe clinical phenotype, which could explain the enrichment in B cells, we observed that the significant association of a B cell–rich synovitis with a clinical phenotype of highly active aggressive RA in untreated early disease was diluted at later stages.

Previous data have suggested that ~40% of RA patients present synovitis characterized by a B cell–rich infiltrate, both in early 9, 10, 11 and late‐stage disease 6. However, the clinical relevance of B cell synovitis has been a subject of controversy, with previous reports presenting conflicting data on the relationship of B cell synovitis with clinical phenotype 15, 16, 17. We attempted to overcome the limitations presented by previous analyses 30, 31 by implementing a consistent and validated semiquantitative B cell score. This score was shown to be a reliable measure of total B cell content by validation against objective histologic and molecular quantitative measurements (digital image analysis and RNA‐Seq transcript lineage analysis, respectively) in large, well‐defined cohorts of patients. Although the validation of semiquantitative histologic synovial T cell and macrophage scores using digital image analysis has been explored previously 32, 33, validation of semiquantitative CD20 scores against both digital image analysis and B cell–lineage transcript analysis has not. In particular, the evaluation against B cell transcripts represents an invaluable confirmation that the semiquantitative histologic score used to grade limited tissue sections accurately reflects the B cell content of the whole specimen (6 additional biopsy specimens pooled for RNA extraction), therefore representing a reliable method for the assessment of B cells in synovia.

The significant association of B cell–rich synovitis with highly active seropositive RA in untreated early disease supports previous observations 12 and suggests that synovial tissue cellular infiltration could help define histologic RA subsets, similarly to what has been described for seronegative and seropositive RA 34. Consistent with these observations, the presence of B cell–rich lymphoid aggregates in early RA has recently been shown to predict treatment response to conventional synthetic DMARDs and radiographic progression 12 and to enhance clinical classification and prognostic/treatment response algorithms 35. In established RA, the associations of B cell synovitis with local inflammation (histologic synovitis) and CRP persisted, and patients showed a more aggressive clinical phenotype overall, but there was a lack of association of B cell–rich synovitis with clinical markers of disease activity such as the DAS28. This lack of association suggests that current standard clinimetric assessment of disease activity may be too insensitive to detect ongoing histologic inflammation, as it might be confounded by factors such as concomitant therapy or comorbidities and their impact on patient‐reported outcomes and disease activity scores. To overcome such limitations, future analyses may include alternative measures of disease activity, including, for example, the reweighted 2‐component imaging‐derived disease activity score 36, and determine their relationship with histologic synovitis. To date, in fact, very little is known about the association of histologic synovitis with clinical phenotypes. Importantly, our observations could provide an explanation for the variable associations observed between clinical phenotype and synovial pathobiology in other disease cohorts where patients with varying disease duration and/or therapies were analyzed together 15, 17.

Such disconnect between ongoing synovial inflammation and clinical disease activity could explain the reported radiographic progression in RA patients with low disease activity treated with synthetic and/or biologic DMARDs 37. However, future analyses, including the outcome of the R4RA trial, will help us to understand whether B cell synovitis continues to drive radiographic progression in late‐stage disease, as recently demonstrated in early disease 12, and to further dissect the association of clinical parameters with histologic synovitis. An alternative explanation for such disconnect could be that different pathogenic mechanisms drive local synovial inflammation in early RA and in established RA and manifest different levels of clinical synovitis. For example, T cell cytokine signatures described in early RA do not appear to be present in later stages 38, while different immune cell infiltration has been found in synovial samples from patients with active RA compared with samples from patients with end‐stage destructive RA obtained at joint replacement 39. Importantly, however, our cohort of patients with established RA did not include patients with end‐stage RA, and the enriched B cell infiltrate in this cohort suggests that adaptive immunity continues to play a pivotal role in active established RA. Nonetheless, alternative non–B cell–driven synovitis may be present in different patients.

This study has some limitations. First, while we have analyzed 2 large cohorts of patients characterized by specific disease stages and drug exposure, our results do not include data on sequential biopsy or longitudinal clinical outcomes. Such data will emerge following completion of the R4RA trial and will be critical to determine whether our observation of an enrichment of B cell synovitis in patients with an inadequate response to TNFi is a treatment effect or is related to a propensity for B cell–rich patients to fail to respond to TNFi therapy.

Second, synovial sampling was performed by both US‐guided methods and arthroscopy, which could be considered a confounding factor due to the heterogeneity of sample retrieval methods and/or patient selection. However, recent data have shown an equivalence in quality outcomes of arthroscopy and US‐guided approaches 40. Moreover, the use of US‐guided biopsy for the majority of the procedures ensured that patients were included in the studies irrespective of joint distribution, a factor that is likely to account for the difference in results compared to previous studies that used only large joint arthroscopy 15, which is known to bias recruitment to patients with more severe disease 41, particularly in early RA.

Finally, we acknowledge that many other immune cells play a role in RA synovitis, driving both disease activity and progression and treatment response 42, 43, 44. We found that B cell infiltration was accompanied by T cells, sublining macrophages, and plasma cells, which is consistent with previously reported data 12. However, when comparing the clinical parameters in the early RA cohort, we observed that higher levels of markers of inflammation were associated with higher scores for all immune cells, but that a higher prevalence of autoantibody positivity was associated exclusively with higher B cell, T cell, and plasma cell scores. Higher disease activity was associated with higher B cell, sublining macrophage, and plasma cell scores. In the cohort of patients with established RA, these additional analyses confirmed the exclusive association of CRP levels with synovitis, without differences in any other parameters, once again suggesting that standard clinimetrics seems unable to pick up ongoing synovitis in established RA.

Overall, these data provide insights into the specific associations of immune cells with clinical phenotypes and highlight the relevance of B cells as markers of ongoing synovitis associated with a clinical phenotype of aggressive disease in early RA. However, alternative histologic scoring systems that aim to more comprehensively assess immune cell infiltration are available, including an integrated histologic scoring system 10, 12 that we have recently developed and that has been verified by combined histologic and ‐omics approaches 11, 45. Although it is likely that in the near future integrated histologic and molecular analyses will identify novel or previously unrecognized pathways mediating treatment response or resistance, in order to translate those studies to routine clinical care there is a need for well‐validated histopathologic scores that can be easily applied for patient classification, such as the B cell synovitis score described herein, particularly if it were demonstrated to have clinical utility for stratifying patients to receive rituximab.

In conclusion, we described the application of a robust, validated B cell synovitis score that closely replicates the quantification of B cells using either digital image analysis or RNA‐Seq analysis and identifies a significant enrichment of B cell synovitis in end‐stage treatment‐resistant RA. Furthermore, we demonstrated a variable association between B cell synovitis and clinical disease activity measurements at different stages of disease progression and drug exposure. In particular, the presence of synovial B cells in established treatment‐resistant RA helps identify patients with ongoing synovial inflammation that is not detected by standard clinimetric assessment. Overall, our study confirms the relevance of synovial B cells in RA and suggests that the classification of patients into B cell rich or B cell poor can contribute to patient stratification.

## AUTHOR CONTRIBUTIONS

All authors were involved in drafting the article or revising it critically for important intellectual content, and all authors approved the final version to be published. Dr. Pitzalis had full access to all of the data in the study and takes responsibility for the integrity of the data and the accuracy of the data analysis.

### Study conception and design

Rivellese, Humby, Pitzalis.

### Acquisition of data

Rivellese, Humby, Fossati‐Jimack, Rizvi, Lliso‐Ribera, Nerviani, Hands, Frias, Thorborn, Jaworska.

### Analysis and interpretation of data

Rivellese, Humby, Bugatti, Lucchesi, Giorli, John, Goldmann, Lewis, Manzo, Bombardieri, Pitzalis.

## Supporting information

Supplementary Figures

Supplementary Table 1

Supplementary Table 2

Supplementary Table 3

Supplementary Table 4

Supplementary Table 5

Supplementary Table 6

## Appendix A THE PEAC‐R4RA INVESTIGATORS

The PEAC‐R4RA investigators are as follows: Iain B. McInnes, Chris Buckley, Peter C. Taylor, Ernest Choy, Arthur Pratt, Christopher Edwards, Maya Buch, Nagui Gendi, Pauline Ho, Bhaskar Dasgupta, Patrick Durez, João Eurico Fonseca, Pier Paolo Sainaghi, Mattia Bellan, John Isaacs, Juan D. Cañete, Alberto Cauli, Mattia Congia, Piero Reynolds, Robert Moots, Nora Ng, Carlomaurizio Montecucco, and Patrick Verschueren.
