# Supplementary material for: B Cell Synovitis and Clinical Phenotypes in Rheumatoid Arthritis: Relationship to Disease Stages and Drug Exposure
Source: Arthritis Rheumatol. 2020 Mar 17;72(5):714–25. doi: 10.1002/art.41184 (PMC7217046; doi:10.1002/art.41184)
Supplement: Supplementary file 1 — Supplementary Figures [file ART-72-714-s001.docx]

**Supplementary Figure 1. Flow chart guiding the assessment of synovial samples.**

**
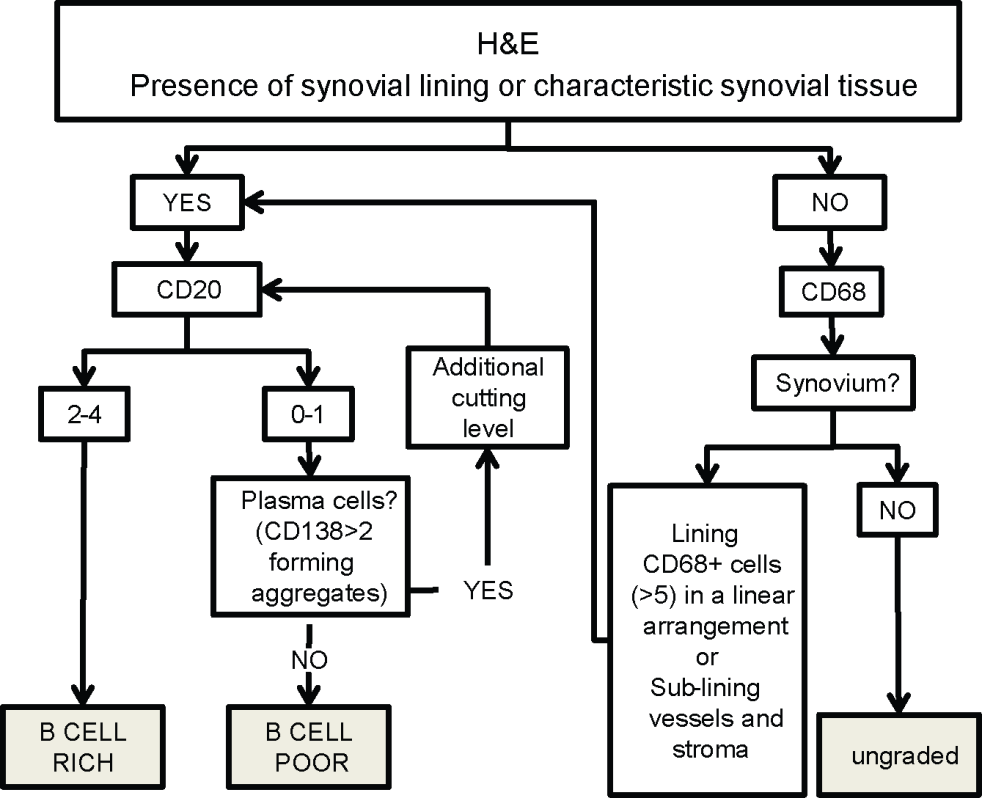
**

The presence of synovial tissue was assessed by H&E and, when necessary, staining with CD68 to look for synovial lining or sub-lining. CD20 staining was then used to classify patients into B cell rich and poor. If B cell poor patients showed CD138+ plasma cell aggregates (score >2) additional cutting levels were assessed

**Supplementary Figure 2. Synovial biopsies cutting protocol**


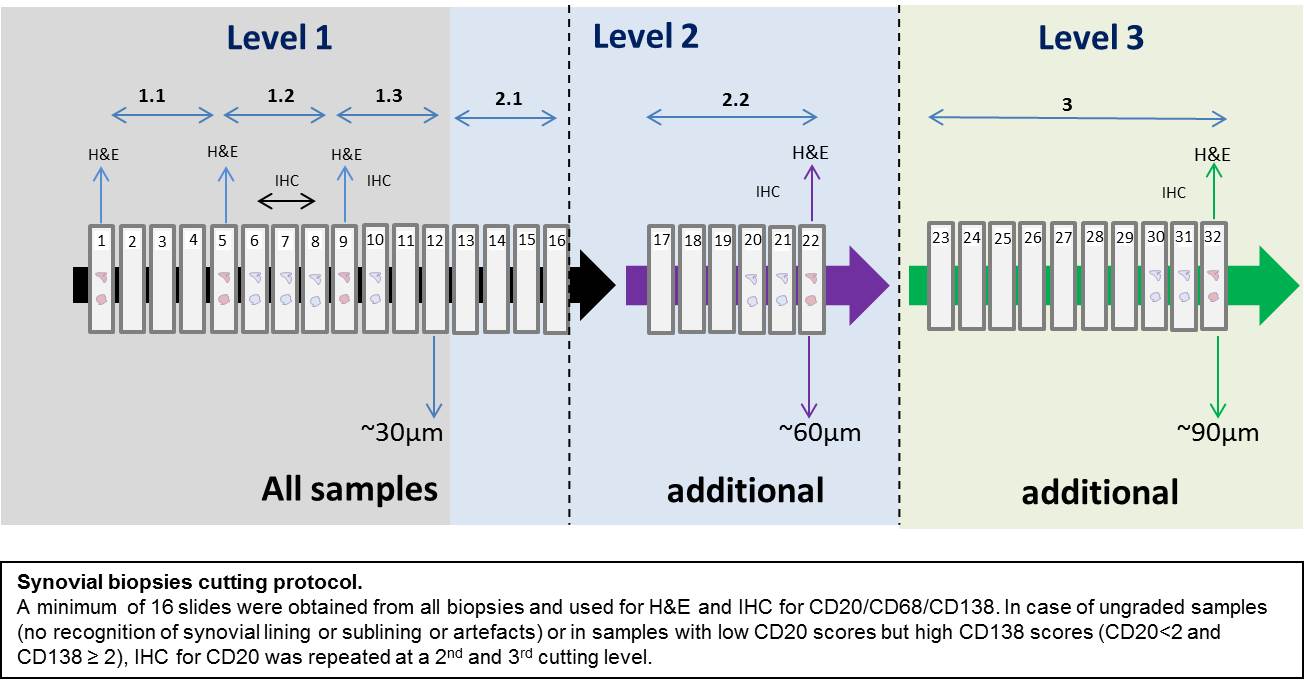


**Supplementary Figure 3. Digital image analysis**

**
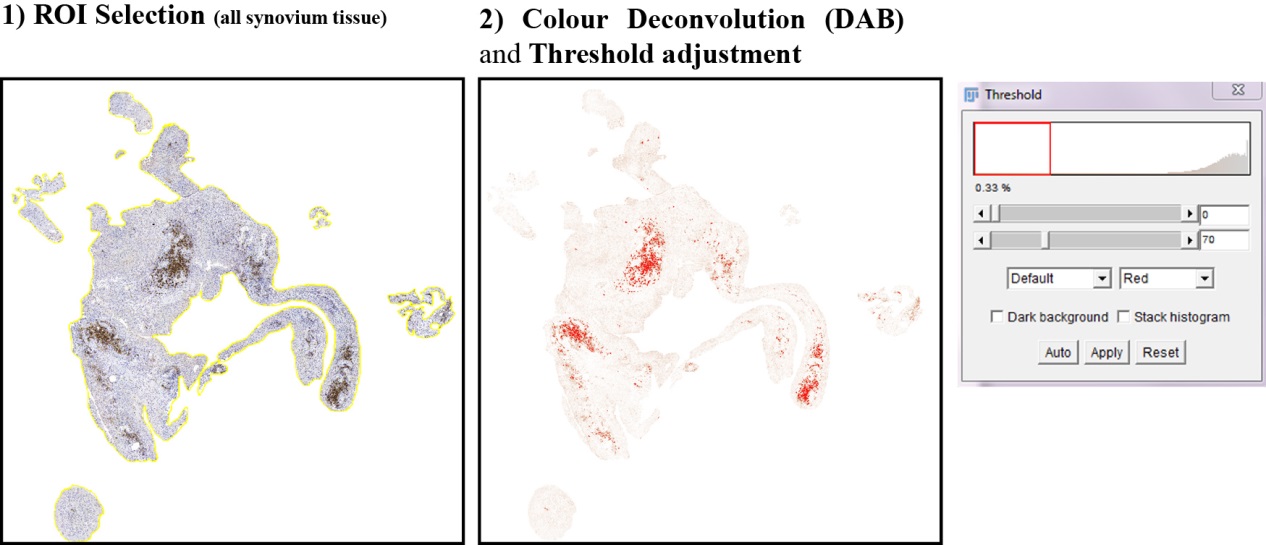
**

Representative image of a patient classified as B cell rich from the TNFi-ir cohort, showing the Region of interest selection (ROI) and the original CD20 + stained area indicated by brown (DAB) staining on tissue (1) and the colour deconvolution and threshold adjustment in (2). The red areas in (2) show the region that the software counts as positive.

The area fraction was calculated as total stained area/total tissue area (ROI) x 100. In the example above total stained area= 213012.77 μm^2^, total tissue area (ROI) = 7419860.27 μm^2^, area fraction 213012.77 / 7419860.27 x 100 = 2.87 %
