# Supplementary material for: B Cell Synovitis and Clinical Phenotypes in Rheumatoid Arthritis: Relationship to Disease Stages and Drug Exposure
Source: Arthritis Rheumatol. 2020 Mar 17;72(5):714–25. doi: 10.1002/art.41184 (PMC7217046; doi:10.1002/art.41184)
Supplement: Supplementary file 2 — Supplementary Table 1 [file ART-72-714-s002.docx]

**Supplementary Table 1.** RNA extraction and sequencing methods of early treatment naïve RA (PEAC) and established TNFi-IR RA (R4RA) cohorts.

|  | | |
| --- | --- | --- |
|  | **Early RA PEAC** | **TNFi-IF (R4RA)** |
| N before filtering | 94 | 128 |
| RNA extraction | Trizol reagent then phenol/chloroform extraction | Trizol reagent then phenol/chloroform (N=4) or column-based extraction (N=124) |
| RNA amount | 1μg of total RNA | 150-500ng of total RNA |
| Library prep | TruSeq RNA Sample Preparation Kit v2 | NEBNext Ultra RNA Library Prep Kit |
| PCR cycles | 10 | 13 |
| Read depth | 50 million | 50 million |
| Read size | 75 base pairs | 150 base pairs |
| Read type | Paired | Paired |
| Sequencing system | Illumina HiSeq 2500 | Illumina HiSeq 4000 |
| Post-QC N for analysis | 91 | 127 |
