# Supplementary material for: B Cell Synovitis and Clinical Phenotypes in Rheumatoid Arthritis: Relationship to Disease Stages and Drug Exposure
Source: Arthritis Rheumatol. 2020 Mar 17;72(5):714–25. doi: 10.1002/art.41184 (PMC7217046; doi:10.1002/art.41184)
Supplement: Supplementary file 3 — Supplementary Table 2 [file ART-72-714-s003.docx]

**Supplementary Table 2.**

| **Supplementary File_B cell module** | | | | | | | |
| --- | --- | --- | --- | --- | --- | --- | --- |
| Cell type | Gene ID | Gene | Peak | Entrezgene | Z score | Specificity | Rank |
| CD19+ B Cells | p1@FCER2 | FCER2 | p1 | 2208 | 17.26031 | 1 | 1 |
| CD19+ B Cells | p2@PNOC | PNOC | p2 | 5368 | 17.08652 | 2 | 1 |
| CD19+ B Cells | p1@CD79B | CD79B | p1 | 974 | 16.67548 | 3 | 1 |
| CD19+ B Cells | p1@IGHM | IGHM | p1 | 3507 | 16.53784 | 3 | 1 |
| CD19+ B Cells | p1@TCL1A | TCL1A | p1 | 8115 | 15.20944 | 3 | 1 |
| CD19+ B Cells | p1@LOC283663 | LOC283663 | p1 | 283663 | 12.13695 | 3 | 1 |
| CD19+ B Cells | p1@IGHD | IGHD | p1 | 3495 | 16.82162 | 4 | 1 |
| CD19+ B Cells | p1@FCRL1 | FCRL1 | p1 | 115350 | 16.41362 | 4 | 1 |
| CD19+ B Cells | p1@FCRL3 | FCRL3 | p1 | 115352 | 16.33682 | 4 | 1 |
| CD19+ B Cells | p4@E2F5 | E2F5 | p4 | 1875 | 16.12247 | 4 | 1 |
| CD19+ B Cells | p1@FAM129C | FAM129C | p1 | 199786 | 12.62951 | 4 | 1 |
| CD19+ B Cells | p2@WDR11 | WDR11 | p2 | 55717 | 8.502624 | 4 | 2 |
| CD19+ B Cells | p1@KIAA0226L | KIAA0226L | p1 | 80183 | 8.003387 | 4 | 3 |
| CD19+ B Cells | p1@IL4R | IL4R | p1 | 3566 | 6.934551 | 4 | 3 |
| CD19+ B Cells | p1@HLA-DPB1 | HLA-DPB1 | p1 | 3115 | 5.744193 | 4 | 3 |
| CD19+ B Cells | p1@BANK1 | BANK1 | p1 | 55024 | 16.38483 | 5 | 1 |
| CD19+ B Cells | p1@MS4A1 | MS4A1 | p1 | 931 | 16.28411 | 5 | 1 |
| CD19+ B Cells | p1@BTLA | BTLA | p1 | 151888 | 15.47911 | 5 | 1 |
| CD19+ B Cells | p1@TMEM156 | TMEM156 | p1 | 80008 | 13.35554 | 5 | 1 |
| CD19+ B Cells | p1@TCL1B | TCL1B | p1 | 9623 | 9.801964 | 5 | 2 |
| CD19+ B Cells | p1@CD79A | CD79A | p1 | 973 | 16.08173 | 6 | 1 |
| CD19+ B Cells | p1@FCRL2 | FCRL2 | p1 | 79368 | 15.32179 | 6 | 1 |
| CD19+ B Cells | p1@RALGPS2 | RALGPS2 | p1 | 55103 | 14.53985 | 6 | 1 |
| CD19+ B Cells | p2@CPNE5 | CPNE5 | p2 | 57699 | 14.379 | 6 | 1 |
| CD19+ B Cells | p2@HTR3A | HTR3A | p2 | 3359 | 12.66723 | 6 | 1 |
| CD19+ B Cells | p1@CD72 | CD72 | p1 | 971 | 12.05548 | 6 | 1 |
| CD19+ B Cells | p1@CD40 | CD40 | p1 | 958 | 8.961206 | 6 | 2 |
| CD19+ B Cells | p1@CLECL1 | CLECL1 | p1 | 160365 | 6.203515 | 6 | 2 |
| CD19+ B Cells | p1@HLA-DRA | HLA-DRA | p1 | 3122 | 5.03172 | 6 | 3 |
| CD19+ B Cells | p1@BLK | BLK | p1 | 640 | 15.45389 | 7 | 1 |
| CD19+ B Cells | p1@HLA-DOB | HLA-DOB | p1 | 3112 | 15.23105 | 7 | 1 |
| CD19+ B Cells | p1@CD22 | CD22 | p1 | 933 | 14.96726 | 7 | 1 |
| CD19+ B Cells | p1@CXCR5 | CXCR5 | p1 | 643 | 14.79641 | 7 | 1 |
| CD19+ B Cells | p1@CLEC17A | CLEC17A | p1 | 388512 | 14.35185 | 7 | 1 |
| CD19+ B Cells | p3@LOC646626 | LOC646626 | p3 | 646626 | 14.33219 | 7 | 1 |
| CD19+ B Cells | p1@CD19 | CD19 | p1 | 930 | 14.31234 | 7 | 1 |
| CD19+ B Cells | p1@ZNF860 | ZNF860 | p1 | 344787 | 14.05719 | 7 | 1 |
| CD19+ B Cells | p1@VPREB3 | VPREB3 | p1 | 29802 | 13.80103 | 7 | 1 |
| CD19+ B Cells | p1@TNFRSF13C | TNFRSF13C | p1 | 115650 | 13.78917 | 7 | 1 |
| CD19+ B Cells | p1@TLR10 | TLR10 | p1 | 81793 | 13.31736 | 7 | 1 |
| CD19+ B Cells | p1@IGLL5 | IGLL5 | p1 | 100423062 | 13.06623 | 7 | 1 |
| CD19+ B Cells | p1@ZBTB32 | ZBTB32 | p1 | 27033 | 11.38486 | 7 | 1 |
| CD19+ B Cells | p4@TLK2 | TLK2 | p4 | 11011 | 11.35922 | 7 | 1 |
| CD19+ B Cells | p1@WDFY4 | WDFY4 | p1 | 57705 | 8.220904 | 7 | 2 |
| CD19+ B Cells | p1@POU2AF1 | POU2AF1 | p1 | 5450 | 7.84268 | 7 | 3 |
| CD19+ B Cells | p1@LCN10 | LCN10 | p1 | 414332 | 7.713228 | 7 | 2 |
| CD19+ B Cells | p1@HLA-DOA | HLA-DOA | p1 | 3111 | 7.594435 | 7 | 2 |
| CD19+ B Cells | p2@RFX5 | RFX5 | p2 | 5993 | 6.811954 | 7 | 2 |
| CD19+ B Cells | p1@CIITA | CIITA | p1 | 4261 | 6.585012 | 7 | 2 |
| CD19+ B Cells | p1@FCRLA | FCRLA | p1 | 84824 | 14.06132 | 8 | 1 |
| CD19+ B Cells | p2@P2RX5 | P2RX5 | p2 | 5026 | 13.71183 | 8 | 1 |
| CD19+ B Cells | p1@LOC400958 | LOC400958 | p1 | 400958 | 12.15307 | 8 | 1 |
| CD19+ B Cells | p2@PAX5 | PAX5 | p2 | 5079 | 11.72762 | 8 | 1 |
| CD19+ B Cells | p1@BACH2 | BACH2 | p1 | 60468 | 11.28966 | 8 | 1 |
| CD19+ B Cells | p1@LINC00494 | LINC00494 | p1 | 284749 | 11.05019 | 8 | 1 |
| CD19+ B Cells | p1@TNFRSF13B | TNFRSF13B | p1 | 23495 | 10.17526 | 8 | 1 |
| CD19+ B Cells | p1@FCRL5 | FCRL5 | p1 | 83416 | 9.792472 | 8 | 1 |
| CD19+ B Cells | p1@SPIB | SPIB | p1 | 6689 | 8.771794 | 8 | 2 |
| CD19+ B Cells | p1@C12orf74 | C12orf74 | p1 | 338809 | 6.508146 | 8 | 2 |
| CD19+ B Cells | p5@FOXP1 | FOXP1 | p5 | 27086 | 5.715134 | 8 | 3 |
| CD19+ B Cells | p1@TAPT1 | TAPT1 | p1 | 202018 | 5.565624 | 8 | 3 |
| CD19+ B Cells | p1@CD74 | CD74 | p1 | 972 | 5.17249 | 8 | 3 |
| CD19+ B Cells | p1@TNFRSF17 | TNFRSF17 | p1 | 608 | 13.82382 | 9 | 1 |
| CD19+ B Cells | p2@PLEKHF2 | PLEKHF2 | p2 | 79666 | 12.58654 | 9 | 1 |
| CD19+ B Cells | p1@COL19A1 | COL19A1 | p1 | 1310 | 12.12973 | 9 | 1 |
| CD19+ B Cells | p1@CD180 | CD180 | p1 | 4064 | 10.60274 | 9 | 1 |
| CD19+ B Cells | p2@FAM177B | FAM177B | p2 | 400823 | 10.45875 | 9 | 1 |
| CD19+ B Cells | p1@BLNK | BLNK | p1 | 29760 | 9.503452 | 9 | 1 |
| CD19+ B Cells | p1@HLA-DMB | HLA-DMB | p1 | 3109 | 7.257757 | 9 | 2 |
| CD19+ B Cells | p1@PPAPDC1B | PPAPDC1B | p1 | 84513 | 5.802356 | 9 | 3 |
| CD19+ B Cells | p1@STAP1 | STAP1 | p1 | 26228 | 14.49539 | 10 | 1 |
| CD19+ B Cells | p3@DUS2L | DUS2L | p3 | 54920 | 13.54725 | 10 | 1 |
| CD19+ B Cells | p1@SNX22 | SNX22 | p1 | 79856 | 10.95506 | 10 | 1 |
| CD19+ B Cells | p1@CNR2 | CNR2 | p1 | 1269 | 10.58818 | 10 | 1 |
| CD19+ B Cells | p1@MARCH1 | MARCH1 | p1 | 55016 | 9.159259 | 10 | 1 |
| CD19+ B Cells | p1@USP6NL | USP6NL | p1 | 9712 | 8.338279 | 10 | 1 |
| CD19+ B Cells | p1@SNX2 | SNX2 | p1 | 6643 | 7.434421 | 10 | 1 |
| CD19+ B Cells | p1@PTPRCAP | PTPRCAP | p1 | 5790 | 6.785584 | 10 | 2 |
| CD19+ B Cells | p1@DRAM2 | DRAM2 | p1 | 128338 | 5.915961 | 10 | 2 |
| CD19+ B Cells | p1@LOC100129196 | LOC100129196 | p1 | 100129196 | 5.847172 | 10 | 3 |
| CD19+ B Cells | p1@STX7 | STX7 | p1 | 8417 | 5.665618 | 10 | 2 |
| CD19+ B Cells | p1@SMC6 | SMC6 | p1 | 79677 | 5.36844 | 10 | 1 |
