# Supplementary material for: B Cell Synovitis and Clinical Phenotypes in Rheumatoid Arthritis: Relationship to Disease Stages and Drug Exposure
Source: Arthritis Rheumatol. 2020 Mar 17;72(5):714–25. doi: 10.1002/art.41184 (PMC7217046; doi:10.1002/art.41184)
Supplement: Supplementary file 4 — Supplementary Table 3 [file ART-72-714-s004.docx]

**Supplementary Table 3. T cells**

|  | | **PEAC (early RA)**  n=140# | | | **R4RA (TNFi-IR)**  n=155# | | |
| --- | --- | --- | --- | --- | --- | --- | --- |
|  | | CD3  SQ score <2  100 (71.4%) | CD3 SQ score ≥ 2  40 (28.6%) | p | CD3  SQ score <2  122 (78.7%) | CD3 SQ score ≥ 2  33 (21.3%) | p |
| **DAS28** mean (SD) | | 5.6 (1.4) | 6 (1.2) | Ns | 5.7 (1.3) | 5.6 (1.3) | ns |
| **TJ** mean (SD) | | 11.6 (7.2) | 12 (7.7) | Ns | 12.4 (8.0) | 9.9 (7.2) | ns |
| **SJ** mean (SD) | | 7.3 (5.4) | 9 (5.7) | ns | 6.9 (5.4) | 7.0 (3.9) | ns |
| **VAS GH**, mean (SD) | | 59.5 (28.7) | 67.6 (24) | 0.016 | 66.1 (23.9) | 65.5 (29.8) | ns |
| **ESR** mean (SD) | | 36.6 (28.9) | 45.9 (25.2) | ns | 34.6 (23.9) | 35.8 (24.2) | ns |
| **CRP** mean (SD) | | 18.6 (32.7) | 22.4 (24.2) | 0.016 | 21.8 (33.9) | 27.4 (28.1) | 0.024 |
| **ACPA**, % | | 60% | 80% | 0.024 | 76.3% | 68.8% | ns |
| **RF+,** % | | 63.6% | 77.5% | 0.114 | 73.3% | 62.5% | ns |
| **csDMARDs**  % | 0 | 100% | 100% | na | 3.3% | 0.0% | ns |
|  | 1 | 0 | 0 |  | 65.6% | 81.8% |  |
|  | 2 | 0 | 0 |  | 23.8% | 15.2% |  |
|  | 3 | 0 | 0 |  | 7.4% | 3.0% |  |
| **Steroids*** % | | 0% | 0% | na | 42% | 36.4% | ns |

#excluding patients with ungraded synovial biopsy samples *Steroids at the time of the biopsy; SQ= semi-quantitative; DAS28 Disease Activity Score 28 joints; TJ Tender Joints; SJ Swollen Joints; VAS GH Visuo-Analogic Score Global Health; ACPA Anti Citrullinated Protein Antibodies measured by clinically available standard path-lab CCP2 assay; RF Rheumatoid Factor: csDMARDs conventional synthetic Disease Modifying Anti-Rheumatic Drugs; CD68L= CD68 Lining; CD68SL= CD68 Sub-lining. na=not applicable; ns=not significant. Mann-Whitney or Fisher tests as appropriate.
