# Supplementary material for: B Cell Synovitis and Clinical Phenotypes in Rheumatoid Arthritis: Relationship to Disease Stages and Drug Exposure
Source: Arthritis Rheumatol. 2020 Mar 17;72(5):714–25. doi: 10.1002/art.41184 (PMC7217046; doi:10.1002/art.41184)
Supplement: Supplementary file 5 — Supplementary Table 4 [file ART-72-714-s005.docx]

**Supplementary Table 4. Lining macrophages**

|  | | **PEAC (early RA)**  n=140# | | | **R4RA (TNFi-IR)**  n=155# | | |
| --- | --- | --- | --- | --- | --- | --- | --- |
|  | | CD68L  SQ score <2  106 (75.7%) | CD68L  SQ score ≥ 2  34 (24.3%) | p | CD68L  SQ score <2  139 (89.7%) | CD68L  SQ score ≥ 2  16 (10.3%) | p |
| **DAS28** mean (SD) | | 5.7(1.4) | 6.1 (1.2) | ns | 5.7 (1.3) | 5.4 (1.3) | ns |
| **TJ** mean (SD) | | 12 (7.5) | 11.9 (7.6) | ns | 12.1 (7.9) | 10.1 (7.6) | ns |
| **SJ** mean (SD) | | 7.3 (5.5) | 9 (7.5) | ns | 6.9 (5.1) | 7.3 (5.3) | ns |
| **VAS GH**, mean (SD) | | 62.3 (28.4) | 62.9 (24.4) | ns | 67.1 (24.1) | 56.9 (32.3) | ns |
| **ESR** mean (SD) | | 36.4 (28.9) | 47.3 (26.8) | 0.017 | 35.3 (26.6) | 30.9 (19.1) | ns |
| **CRP** mean (SD) | | 19.5 (30.9) | 18.1 (17) | ns | 22.6  (32.2) | 26.5 (30.3) | ns |
| **ACPA**, % | | 66% | 64.7% | ns | 76.1% | 62.5% | ns |
| **RF+,** % | | 66.7% | 70.6% | ns | 71.5% | 66.7% | ns |
| **csDMARDs**  % | 0 | 100% | 100% | na | 2.9% | 0.0% | ns |
|  | 1 | 0 | 0 |  | 66.2% | 93.8% |  |
|  | 2 | 0 | 0 |  | 23.7% | 6.3% |  |
|  | 3 | 0 | 0 |  | 13.5% | 0.0% |  |
| **Steroids*** % | | 0% | 0% | na | 40.4% | 43.8% | ns |

#excluding patients with ungraded synovial biopsy samples *Steroids at the time of the biopsy; SQ= semi-quantitative; DAS28 Disease Activity Score 28 joints; TJ Tender Joints; SJ Swollen Joints; VAS GH Visuo-Analogic Score Global Health; ACPA Anti Citrullinated Protein Antibodies measured by clinically available standard path-lab CCP2 assay; RF Rheumatoid Factor: csDMARDs conventional synthetic Disease Modifying Anti-Rheumatic Drugs; CD68L= CD68 Lining; CD68SL= CD68 Sub-lining. na=not applicable; ns=not significant. Mann-Whitney or Fisher tests as appropriate.
