# Supplementary material for: B Cell Synovitis and Clinical Phenotypes in Rheumatoid Arthritis: Relationship to Disease Stages and Drug Exposure
Source: Arthritis Rheumatol. 2020 Mar 17;72(5):714–25. doi: 10.1002/art.41184 (PMC7217046; doi:10.1002/art.41184)
Supplement: Supplementary file 6 — Supplementary Table 5 [file ART-72-714-s006.docx]

**Supplementary Table 5. Sub-lining macrophages**

|  | | **PEAC (early RA)**  n=140# | | | **R4RA (TNFi-IR)**  n=155# | | |
| --- | --- | --- | --- | --- | --- | --- | --- |
|  | | CD68SL  SQ score <2  106 (75.7%) | CD68SL  SQ score ≥ 2  34 (24.3%) | p | CD68SL  SQ score <2  120 (77.4%) | CD68SL  SQ score ≥ 2  35 (22.6%) | p |
| **DAS28** mean (SD) | | 5.5(1.4) | 6.1 (1.2) | 0.009 | 5.7 (1.3) | 5.6 (1.0) | ns |
| **TJ** mean (SD) | | 11.6 (7.6) | 12.1 (7.4) | Ns | 12.5 (8.2) | 9.7 (6.3) | ns |
| **SJ** mean (SD) | | 7.2 (5.5) | 8.3 (5.6) | ns | 6.7 (4.9) | 7.7 (5.7) | ns |
| **VAS GH**, mean (SD) | | 59.1 (29.2) | 66.1 (24.7) | ns | 65.4 (24.8) | 68.0 (26.6) | ns |
| **ESR** mean (SD) | | 30.5 (24.7) | 49.4 (30) | <0.001 | 33.9 (25.4) | 38.1 (27.8) | ns |
| **CRP** mean (SD) | | 14.2 (30.2) | 26 (25.7) | <0.001 | 18.8 (26.8) | 37.3 (42.8) | 0.001 |
| **ACPA**, % | | 63.6% | 70.3% | ns | 78.3% | 62.9% | ns |
| **RF+,** % | | 62.3% | 74.6% | ns | 72.9% | 64.7% | ns |
| **csDMARDs**  % | 0 | 100% | 100% | na | 2.5% | 2.9% | ns |
|  | 1 | 0 | 0 |  | 65.0% | 82.9% |  |
|  | 2 | 0 | 0 |  | 24.2% | 14.3% |  |
|  | 3 | 0 | 0 |  | 8.3% | 0.0% |  |
| **Steroids*** % | | 0% | 0% | na | 39.3% | 45.7% | ns |

#excluding patients with ungraded synovial biopsy samples *Steroids at the time of the biopsy; SQ= semi-quantitative; DAS28 Disease Activity Score 28 joints; TJ Tender Joints; SJ Swollen Joints; VAS GH Visuo-Analogic Score Global Health; ACPA Anti Citrullinated Protein Antibodies measured by clinically available standard path-lab CCP2 assay; RF Rheumatoid Factor: csDMARDs conventional synthetic Disease Modifying Anti-Rheumatic Drugs; CD68L= CD68 Lining; CD68SL= CD68 Sub-lining. na=not applicable; ns=not significant. Mann-Whitney or Fisher tests as appropriate.
