# Supplementary material for: B Cell Synovitis and Clinical Phenotypes in Rheumatoid Arthritis: Relationship to Disease Stages and Drug Exposure
Source: Arthritis Rheumatol. 2020 Mar 17;72(5):714–25. doi: 10.1002/art.41184 (PMC7217046; doi:10.1002/art.41184)
Supplement: Supplementary file 7 — Supplementary Table 6 [file ART-72-714-s007.docx]

**Supplementary Table 6. Plasma Cells**

|  | | **PEAC (early RA)**  n=140# | | | **R4RA (TNFi-IR)**  n=155# | | |
| --- | --- | --- | --- | --- | --- | --- | --- |
|  | | CD138  SQ score <2  98 (69.5%) | CD138  SQ score ≥ 2  43 (30.5%) | p | CD138  SQ score <2  112 (72.6%) | CD138  SQ score ≥ 2  43 (27.7%) | p |
| **DAS28** mean (SD) | | 5.6 (1.4) | 6.1 (1.2) | 0.014 | 5.6 (1.3) | 5.7 (1.2) | ns |
| **TJ** mean (SD) | | 11.7 (7.6) | 12.2 (7.4) | ns | 12.2 (7.8) | 11.1 (8.1) | ns |
| **SJ** mean (SD) | | 7.6 (5.6) | 8.3 (7.0) | ns | 6.7 (5.2) | 7.5 (4.9) | ns |
| **VAS GH**, mean (SD) | | 60.3 (28.8) | 68.2 (22.0) | ns | 65.4 (24.8) | 67.5 (26.4) | ns |
| **ESR** mean (SD) | | 33.2 (26.2) | 53.7 (29.1) | <0.001 | 34.4 (26.5) | 36.1 (24.6) | ns |
| **CRP** mean (SD) | | 17.4 (29.9) | 24.8 (25.5) | 0.07 | 20.7 (30.2) | 29.9 (35.6) | 0.005 |
| **ACPA**, % | | 57.1% | 88.4% | <0.001 | 74.1% | 76.2% | ns |
| **RF+,** % | | 58.2% | 92.9% | <0.001 | 69.1% | 76.2% | ns |
| **csDMARDs**  % | 0 | 100% | 100% | na | 1.8% | 4.7% | ns |
|  | 1 | 0 | 0 |  | 65.2% | 79.1% |  |
|  | 2 | 0 | 0 |  | 26.8% | 9.3% |  |
|  | 3 | 0 | 0 |  | 6.3% | 7.0% |  |
| **Steroids*** % | | 0% | 0% | na | 37.6% | 48.8% | ns |

#excluding patients with ungraded synovial biopsy samples *Steroids at the time of the biopsy; SQ= semi-quantitative; DAS28 Disease Activity Score 28 joints; TJ Tender Joints; SJ Swollen Joints; VAS GH Visuo-Analogic Score Global Health; ACPA Anti Citrullinated Protein Antibodies measured by clinically available standard path-lab CCP2 assay; RF Rheumatoid Factor: csDMARDs conventional synthetic Disease Modifying Anti-Rheumatic Drugs; CD68L= CD68 Lining; CD68SL= CD68 Sub-lining. na=not applicable; ns=not significant. Mann-Whitney or Fisher tests as appropriate.
